# ﻿*Shuqiangius* gen. nov., a new genus of Oxyopidae (Arachnida, Araneae) from China

**DOI:** 10.3897/zookeys.1261.171511

**Published:** 2025-11-28

**Authors:** Ying Wang, Qingzhen Meng, Yuri M. Marusik, Zhiyuan Yao

**Affiliations:** 1 College of Life Science, Shenyang Normal University, Shenyang 110034, Liaoning, China Shenyang Normal University Shenyang China; 2 Institute for Biological Problems of the North FEB RAS, Portovaya Str. 18, Magadan 685000, Russia Institute for Biological Problems of the North FEB RAS Magadan Russia; 3 Altai State University, Lenina Pr., 61, Barnaul, RF-656049, Russia Altai State University Barnaul Russia; 4 Department of Zoology & Entomology, University of the Free State, Bloemfontein 9300, South Africa University of the Free State Bloemfontein South Africa

**Keywords:** Aranei, Asia, lynx spider, new combination, new species

## Abstract

A new genus *Shuqiangius* Wang, Marusik & Yao, **gen. nov.** is described. It comprises the type species *Shuqiangius rarobulbus* (Lo, Cheng & Lin, 2024), **comb. nov.** (♂; Taiwan, China) and a new species *Shuqiangius chuan* Wang, Marusik & Yao, **sp. nov.** (♂♀; Sichuan, China).

## ﻿Introduction

Oxyopidae is a relatively small family with 448 extant species belonging to nine genera (WSC 2025). The most speciose genus of this family is *Oxyopes* Latreille, 1804, including 284 valid species (WSC 2025). There has not been any global revision of the family, nor has it been split into subfamilies. The family is poorly studied in China and adjacent regions. All that is known is that the numbers of species recorded in China comprise 34 species of *Oxyopes* (Schenkel 1936; Hu and Wu 1989; Song et al. 1999; Zhang and Zhu 2005; Zhang et al. 2005a, 2005b; Esyunin et al. 2011; Tang and Li 2012; Yin et al. 2012), two species of *Hamadruas* Deeleman-Reinhold, 2009 (Hu et al. 1983; Hu and Zhang 1984; Song 1991; Tang and Li 2012), 18 species of *Hamataliwa* Keyserling, 1887 (Zhang et al. 2005c; Tang and Li 2012; Tang et al. 2012; Lin et al. 2022; Lo et al. 2024a, 2024b), three species of *Peucetia* Thorell, 1869 (Kayashima 1939; Hu et al. 1987), and four species of *Tapponia* Simon, 1885 (Lo et al. 2024a, 2024b).

While studying spiders from Sichuan, China, we found a species that is somatically similar to *Oxyopes* but having a combination of genitalic characters not seen in *Oxyopes* and other members of Oxyopidae, except for one from Taiwan, which has hitherto been described under the genus *Tapponia*. Both species (from Taiwan and Sichuan) are clearly related and should be placed in a new genus. The goal of this paper is to characterize the new genus and provide a description of the new species from Sichuan.

## ﻿Material and methods

Spider specimens were examined and measured with a Leica M205 C stereomicroscope. Left male palp was photographed. Epigynes were photographed before dissection. Epigynes were previously treated in a 10% warm solution of potassium hydroxide (KOH) to dissolve soft tissues before illustration of the endogyne. The majority of the setae on the male palp’s cymbium were removed to facilitate the observation of fine structures, resulting in their absence in the illustrations. Images were captured with a Canon EOS 750D wide zoom digital camera (24.2 megapixels) mounted on the stereomicroscope mentioned above, and assembled using Helicon Focus v. 3.10.3 image stacking software (Khmelik et al. 2005). All measurements are given in millimeters (mm). The measurements of the palp and legs are given as the total length (palp: femur, patella, tibia, and tarsus lengths; leg: femur, patella, tibia, metatarsus, and tarsus lengths). Leg segments were measured on their dorsal side. The distribution map was generated with ArcGIS v. 10.2 (ESRI Inc.). The specimens studied are preserved in 75% ethanol and deposited in the Shenyang Normal University in Liaoning, China.

The abbreviations followed Tang and Li (2012) with some modifications: **AER** = anterior eye row; **ALE** = anterior lateral eye; **ALE–PLE** = distance between ALE and PLE; **AME** = anterior median eye; **AME–ALE** = distance between AME and ALE; **AME–AME** = distance between AMEs; **d** = dorsal; **Fe** = femur; **Mt** = metatarsus; **p** = prolateral; **Pa** = patella; **PER** = posterior eye row; **PLE** = posterior lateral eye; **PME** = posterior median eye; **PME–PLE** = distance between PME and PLE; **PME-PME** = distance between PMEs; **r** = retrolateral; **Ta** = tarsus; **Ti** = tibia; **TL** = tegular lobe; **v** = ventral.

## ﻿Taxonomy

**﻿Family Oxyopidae Thorell, 1869**

### ﻿Genus Shuqiangius

Taxon classificationAnimaliaAraneaeOxyopidae

Wang, Marusik & Yao gen. nov.

D75DC6E4-275C-5C6E-A683-F9CC76914E7B

https://zoobank.org/FA59DAA5-7FBA-45A4-AAF1-1FC1A2B9A165

#### Type species.

*Shuqiangius rarobulbus* (Lo, Cheng & Lin, 2024), comb. nov.

#### Etymology.

The generic name is dedicated to Prof. Shuqiang Li (Anhui, China) who has contributed much to our understanding of spider taxonomy. A masculine noun in nominative case.

#### Diagnosis.

The new genus resembles *Hamadruas* Deeleman-Reinhold, 2009 (Deeleman-Reinhold 2009) by having similar embolus (E) forming 360° loop and terminating almost in center of bulb, and round spermatheca (S), but can be distinguished by the tip of cymbium longer than wide (vs wider than long) and by the presence of distinct posterior pocket (PP; vs absent). Males of this new genus can be distinguished from those of all other genera except for *Peucetia* Thorell, 1869 by the tip of cymbium longer than wide (vs wider than long or as long as wide). Males of this new genus can be distinguished from those of *Peucetia* by the absence of paracymbium (vs present). Females can be distinguished from those of other genera (including *Peucetia*) by having a long epigynal atrium ca 1.5× longer than wide (vs absent or wider than long), distinct posterior pocket (vs lacking), atrium with a thin, septum-like stripe of setae centrally and more numerous posteriorly (vs setae lacking or not documented) (Blackwall 1858; Simon 1866, 1885, 1898a, 1898b; Keyserling 1887; Thorell 1895; Rainbow 1915; Brady 1964; Deeleman-Reinhold 2004, 2009).

#### Description.

**Male**: Total length 3.70–4.35. Carapace egg-shaped, length/width ratio 1.16 or 1.29, brownish or yellowish brown, covered with white setae. Radial furrows indistinct and fovea longitudinal. In dorsal view, eyes with AER strongly recurved and PER strongly procurved (Fig. 2A). Chelicerae brown or yellowish brown, with one promarginal and one retromarginal teeth. Endites yellowish or dark brown. Sternum brown or dark brown, length/width ratio ca 1.28. Legs yellowish, clothed with several long spines on femur, patella, tibia, and metatarsus (Fig. 2A, B). Leg formula: I > II > III > IV. Abdomen oval, covered dense, white setae (Fig. 2A, B). Cardiac mark indistinct (Fig. 2A, B).

Palp as in Figs 3A–D, 4A–C; femur length/width ratio 3.43; patella length/width 0.28/0.21; tibia wider than long, with three apophyses. Retrolateral tibial apophysis (RTA) large and twisted; ventral tibial apophysis (VTA) digitiform or pointed. Tip of cymbium as long as wide or longer than wide. Tegular lobe (TL) small/lacking. Conductor (C) long and wide, with membranous base, tip bend and point toward proximal. Embolus (E) very long forming 360° loop and terminating almost in center of bulb, base of embolus (E) large located in retrolateral half of bulb.

**Female**: Total length 4.90–5.77. Carapace egg-shaped, length/width ratio 1.19–1.24, brown, covered with white setae, except for medially. Sternum length/width ratio 1.19–1.21. Abdomen from light to almost uniformly brown; venter with broad, dark, median band from epigastric furrow to spinnerets, and light yellowish bands along median band (Fig. 5A–F).

Epigynes and Endogynes as in Fig. 6A–F; epigynal plate about as wide as long, with large entirely rebordered atrium, 1.5 times longer than wide, widest in anterior 1/5; posteriorly with distinct pocket (PP); medially atrium with kind of septum (not rebordered, just raised median part). Copulatory openings (CO) anteriorly situated. Copulatory ducts (CD) curved. Fertilization ducts (FD) slender with hook-like terminal.

#### Distribution.

**Figure 1.**
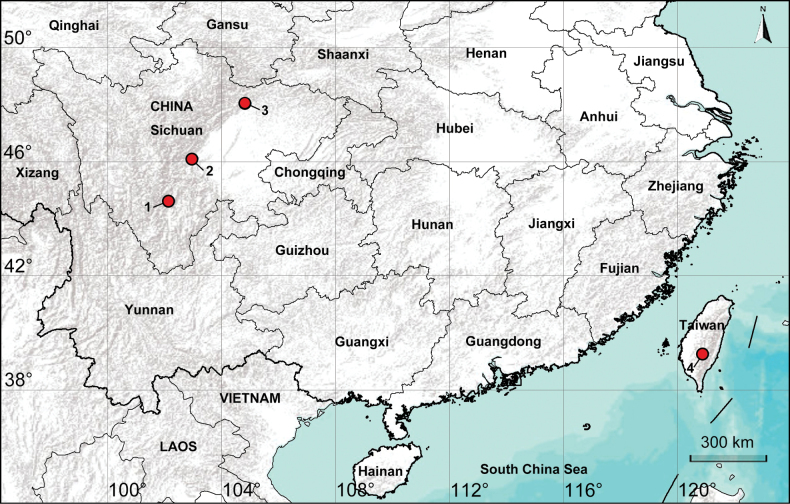
Distribution records of the genus *Shuqiangius* gen. nov. 1–3: *Shuqiangius chuan* sp. nov. 4: *Shuqiangius rarobulbus* comb. nov.

China (Sichuan; Taiwan, type locality; Fig. 1).

#### Composition.

*S. rarobulbus* (Lo, Cheng & Lin, 2024), comb. nov. and *S. chuan* Wang, Marusik & Yao, sp. nov.

### ﻿ Shuqiangius rarobulbus

Taxon classificationAnimaliaAraneaeOxyopidae

(Lo, Cheng & Lin, 2024) comb. nov.

12CD9109-3739-5A6B-B4B1-3330077EBB78

Tapponia rarobulbus Lo et al. 2024a: 37, figs 25A–D, 26A–C (♂, nomen nudum).
Tapponia rarobulbus Lo et al. 2024b: 2 (♂, validation of species description).

#### Type material.

Not examined.

#### Diagnosis.

This species is similar to *S. chuan* sp. nov. but easily distinguished by a male palp with small tegular lobe (TL, Lo et al. 2024a: figs 25B, 26B vs lacking, Figs 3C, 4A–C), by tip of cymbium ca 1/3 of overall length of cymbium (Lo et al. 2024a: figs 25B–D, 26A–C vs 1/2, Fig. 3A–D), by tip of cymbium blunt (Lo et al. 2024a: figs 25B–D, 26A–C vs sharply pointed, Fig. 3A–C), by base of conductor (C) weakly sclerotized and brown (Lo et al. 2024a: fig. 25B vs transparent, Figs 3A–C, 4B–C), and by retrolateral tibial apophysis (RTA) ca 1/2 of tibial length (figs 25B–D, 26A–C in Lo et al. 2024a vs 1/3, Fig. 3A–D).

#### Description.

See Lo et al. (2024a, 2024b).

#### Distribution.

China (Taiwan, type locality; Fig. 1).

### ﻿ Shuqiangius chuan

Taxon classificationAnimaliaAraneaeOxyopidae

Wang, Marusik & Yao sp. nov.

45021F01-7050-58FE-B3F8-B06AC7C77682

https://zoobank.org/FE6F540-277E-451A-9B05-978FF0356B8B

Figs 2, 3, 4, 5, 6

#### Type material.

***Holotype***: China • ♂; Sichuan, Jiangyou Co., Chonghua Town, Wuma Rd; 32.0259°N, 104.9275°E; elev. 796 m; 15 May 2024; X. Zhang, Y. Wang & Q. Meng leg.; SYNU-Ar00496. ***Paratypes***: China • 2♀; same data as for holotype; SYNU-Ar00497–98 • 1♂; Sichuan, Lushan Co., Feixianguan Town, Longdongpo Vill.; 30.0900°N, 103.0675°E; elev. 905 m; 24 May 2024; X. Zhang, Y. Wang & Q. Meng leg.; SYNU-Ar00499 • 1♀; same data as for preceding; SYNU-Ar00500 • 1♀; Sichuan, Liangshan Yi Aut. Pref., Mianning Co., Yihai Town, Damawu Vill.; 28.6107°N, 102.2369°E; elev. 2213 m; 9 Jun 2024; X. Zhang, Y. Wang & Q. Meng leg.; SYNU-Ar00501.

#### Etymology.

The specific name is derived from the type locality (Sichuan) and is a noun in apposition.

#### Diagnosis.

The new species is similar to *S. rarobulbus* comb. nov., but can be easily distinguished by a male palp lacking tegular lobe (TL, Figs 3C, 4A–C vs small, Lo et al. 2024a: figs 25B, 26B), by tip of cymbium ca 1/2 of overall length of cymbium (Fig. 3A–D vs 1/3, Lo et al. 2024a: figs 25B–D, 26A–C), by tip of cymbium sharply pointed (Fig. 3A–C vs blunt, Lo et al. 2024a: figs 25B–D, 26A–C), by base of conductor (C) transparent (Figs 3A–C, 4B–C vs weakly sclerotized and brown, Lo et al. 2024a: fig. 25B), and by retrolateral tibial apophysis (RTA) ca 1/3 of tibial length (Fig. 3A–D vs 1/2, Lo et al. 2024a: figs 25B–D, 26A–C).

#### Description.

**Figure 2.**
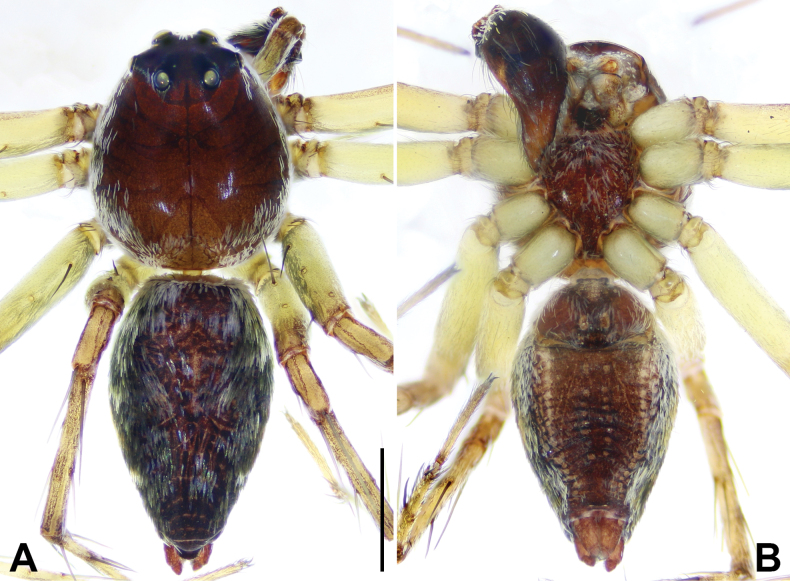
*Shuqiangius chuan* sp. nov., holotype male , habitus. **A.** Dorsal view; **B.** Ventral view. Scale bar: 1 mm (**A, B**).

**Male (holotype)**: Total length 4.35; carapace length/width 1.85/1.59; abdomen length/width 2.50/1.33. Carapace egg-shaped, brownish, covered with white setae. Radial furrows indistinct and fovea longitudinal. In dorsal view, eyes with AER strongly recurved and PER strongly procurved. Ocular area blackish brown with dense setae (Fig. 2A). Eye diameters and inter-distances: AME 0.08, ALE 0.22, PME 0.16, PLE 0.15, AME–AME 0.09, AME–ALE 0.06, PME–PLE 0.24, ALE–PLE 0.15, PME–PME 0.24; eye sizes ALE > PME > PLE > AME. Clypeus height 0.38. Chelicerae yellowish brown, with 1 promarginal and 1 retromarginal teeth. Endite dark brown. Sternum dark brown, length/width 1.00/0.78. Legs yellowish (Fig. 2A, B). Measurements of palp and legs: palp 2.67 (0.70, 0.30, 0.32, 1.35), leg I 8.23 (2.25, 0.72, 2.20, 2.03, 1.03), leg II 7.81 (2.13, 0.70, 2.03, 2.00, 0.95), leg III 6.48 (1.88, 0.63, 1.54, 1.68, 0.75), leg IV 6.04 (1.70, 0.61, 1.33, 1.65, 0.75). Abdomen oval, dorsally dark brown, with yellow markings; laterally with yellow pattern; ventrally brown with yellow dashed spots nearly symmetrically distributed in the middle and at edges. Cardiac mark indistinct (Fig. 2A, B).

**Figure 3.**
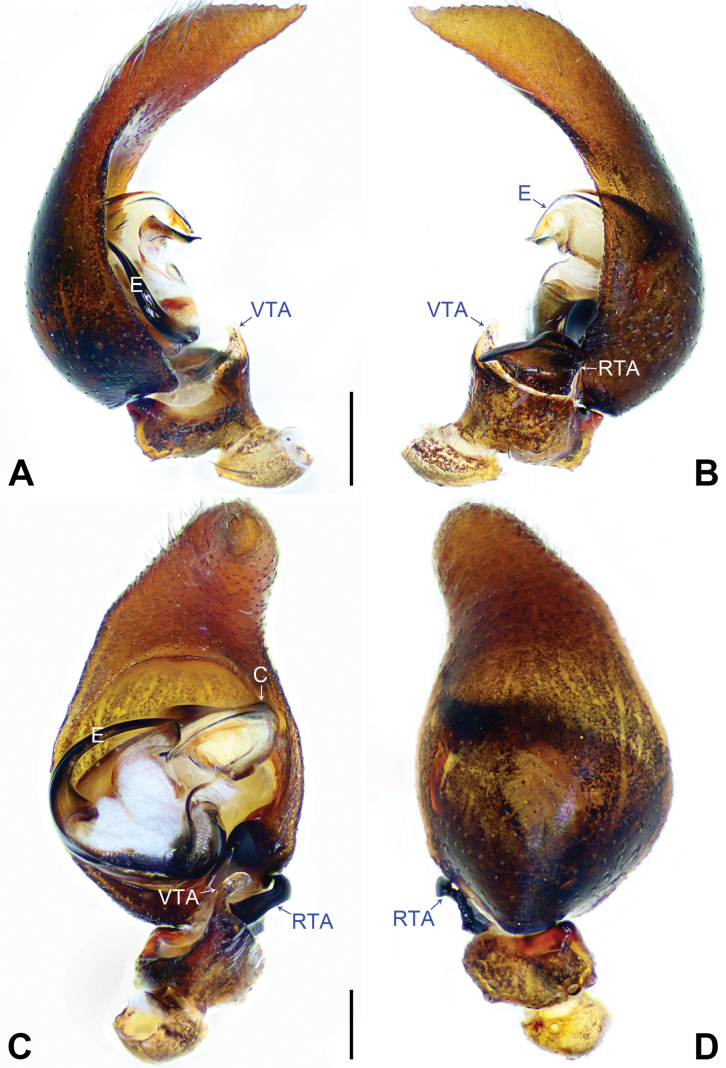
*Shuqiangius chuan* sp. nov., holotype male, palp. **A.** Prolateral view; **B.** Retrolateral view; **C.** Ventral view; **D.** Dorsal view. C = conductor, E = embolus, RTA = retrolateral tibial apophysis, VTA = ventral tibial apophysis. Scale bars: 0.2 mm (**A–D**).

Palp as in Fig. 3A–D; femur length/width 0.72/0.21; patella length/width 0.28/0.21; tibia wider than long, with three apophyses. Retrolateral tibial apophysis (RTA) large and twisted; ventral tibial apophysis (VTA) digitiform or pointed; cymbium longer than femur, patella and tibia together; ca 2.7 times longer than wide, tip of cymbium very long ca 1/2 of cymbium length and longer than wide. Bulb ca 1.3 times wider than long. Tegular lobe (TL) lacking. Conductor (C) long and wide, with membranous base, tip bend and point toward proximal. Embolus (E) very long forming 360° loop and terminating almost in center of bulb, base of embolus (E) large located in retrolateral half of bulb.

**Figure 4.**
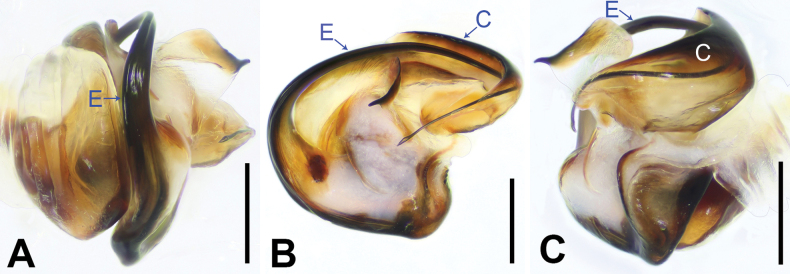
*Shuqiangius chuan* sp. nov., paratype male, bulb. **A.** Prolateral view; **B.** Ventral view; **C.** Retrolateral view. C = conductor, E = embolus. Scale bars: 0.2 mm (**A–C**).

Male leg spination:

**Table:** 

	Fe	Pa	Ti	Mt
I	2d 2p 2r	2d 1r	3d 3p 2r 2v	3d 4p 5r 1v
II	3d 1p 2r	2d 2r	2d 2p 2r 4v	4d 4p 4r
III	2d 1p 1r	2d 1r	2d 2p 2r 4v	4d 3p 2r 5v
IV	2d	1d	2d 2p 1r 2v	4d 4p 3r 2v

**Figure 5.**
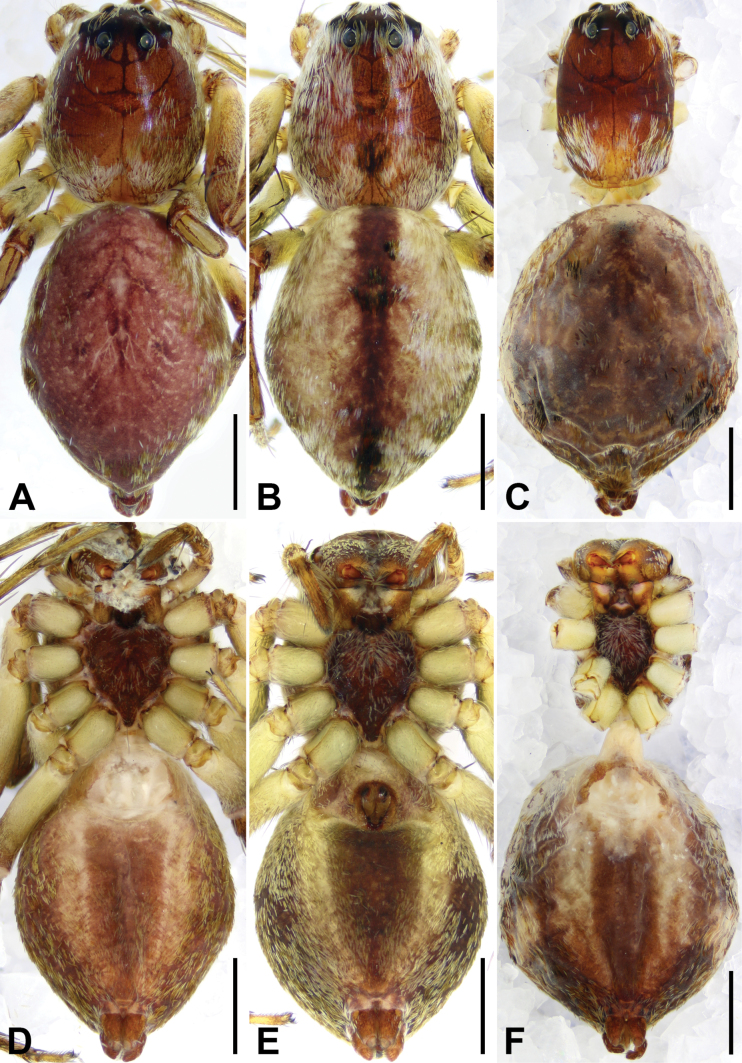
*Shuqiangius chuan* sp. nov., paratype females, habitus. **A–C.** Dorsal view; **D–F.** Ventral view. Scale bars: 1 mm (**A–F**).

**Female** (**paratype**, SYNU-Ar00497): Total length 5.15; carapace length/width 2.16/1.81; abdomen length/width 2.99/2.32. Carapace brown, covered with dense white setae, except for medially. Eye diameters and inter-distances: AME 0.06, ALE 0.20, PME 0.17, PLE 0.16, AME–AME 0.13, AME–ALE 0.06, PME–PLE 0.25, ALE–PLE 0.18, PME–PME 0.30; eye sizes ALE > PME > PLE > AME. Clypeus height 0.51. Sternum length/width 1.05/0.88. Measurements of palps and legs: palp 2.12 (0.59, 0.34, 0.46, 0.73), leg I 7.72 (2.33, 0.76, 1.98, 1.66, 0.99), leg II 7.12 (2.13, 0.74, 1.80, 1.56, 0.89), leg III 5.93 (1.82, 0.65, 1.30, 1.40, 0.76), leg IV 5.64 (1.68, 0.64, 1.20, 1.38, 0.74). Abdomen light yellow, with brown median band, sides darker; venter with broad, dark, median band from epigastric furrow to spinnerets, and light yellowish bands along median band (Fig. 5B, E).

**Figure 6.**
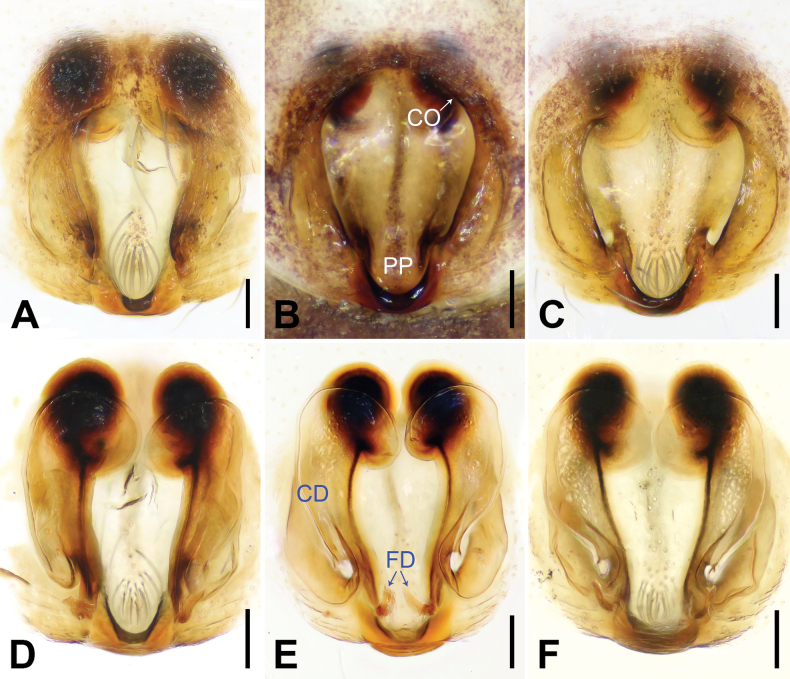
*Shuqiangius chuan* sp. nov., paratype females. **A–C.** Epigynes, ventral view; **D–F.** Endogynes, dorsal view. CD = copulatory duct, CO = copulatory opening, FD = fertilization duct, PP = posterior pocket. Scale bars: 0.1 mm (**A–F**).

Epigyne and endogyne as in Fig. 6B, E; epigynal plate about as wide as long, with large entirely rebordered atrium, 1.5 times longer than wide, widest in anterior 1/5; posteriorly with distinct pocket (PP); medially atrium with kind of septum (not rebordered, just raised median part). Copulatory openings (CO) anteriorly situated. Copulatory ducts (CD) curved. Fertilization ducts (FD) slender with hook-like terminal.

Female leg spination:

**Table:** 

	Fe	Pa	Ti	Mt
I	2d 2p 2r	2d 1p 1r	2d 3p 2r 4v	4d 3p 3r 5v
II	2d 1p 2r	1d 1r 1p	4d 2p 2r 4v	4d 3p 3r 5v
III	2d 1p 1r	2d 1r	2d 2p 2r 4v	4d 3p 3r 5v
IV	3d 1p 1r	2d 1r	2d 1p 1r 2v	6d 1p 1r 5v

#### Variation.

SYNU-Ar00498, 500–501: Total length 4.90, 5.33, 5.77; carapace length 2.10, 2.13, 2.14. Abdomen from light to almost uniformly brown (Fig. 5A, C, D, F).

#### Distribution.

China (Sichuan, type locality; Fig. 1).

## Supplementary Material

XML Treatment for Shuqiangius

XML Treatment for Shuqiangius rarobulbus

XML Treatment for Shuqiangius chuan

## ﻿Additional information

### Use of AI

No use of AI was reported.

### Author ORCIDs

Ying Wang https://orcid.org/0009-0007-9173-4438

Qingzhen Meng https://orcid.org/0009-0009-1143-676X

Yuri M. Marusik https://orcid.org/0000-0002-4499-5148

Zhiyuan Yao https://orcid.org/0000-0002-1631-0949

### Data availability

All of the data that support the findings of this study are available in the main text.
